# Carbon Nanomaterials (CNMs) and Enzymes: From Nanozymes to CNM-Enzyme Conjugates and Biodegradation

**DOI:** 10.3390/ma15031037

**Published:** 2022-01-28

**Authors:** Petr Rozhin, Jada Abdel Monem Gamal, Silvia Giordani, Silvia Marchesan

**Affiliations:** 1 Department of Chemical and Pharmaceutical Sciences, University of Trieste, 34127 Trieste, Italy; petr.rozhin@phd.units.it; 2 School of Chemical Sciences, Faculty of Science & Health, Dublin City University, D09 E432 Dublin, Ireland; jada.abdelmonemgamal2@mail.dcu.ie; 3 Department of Chemistry, Faculty of Mathematical, Physical and Natural Sciences, University Sapienza of Rome, 00185 Rome, Italy

**Keywords:** carbon nanomaterials, carbon nanotubes, graphene, carbon nano-onions, carbon nanodots, enzymes, nanozymes, sensing, fuel cells, medicine

## Abstract

Carbon nanomaterials (CNMs) and enzymes differ significantly in terms of their physico-chemical properties—their handling and characterization require very different specialized skills. Therefore, their combination is not trivial. Numerous studies exist at the interface between these two components—especially in the area of sensing—but also involving biofuel cells, biocatalysis, and even biomedical applications including innovative therapeutic approaches and theranostics. Finally, enzymes that are capable of biodegrading CNMs have been identified, and they may play an important role in controlling the environmental fate of these structures after their use. CNMs’ widespread use has created more and more opportunities for their entry into the environment, and thus it becomes increasingly important to understand how to biodegrade them. In this concise review, we will cover the progress made in the last five years on this exciting topic, focusing on the applications, and concluding with future perspectives on research combining carbon nanomaterials and enzymes.

## 1. Introduction

Carbon nanomaterials (CNMs) and enzymes belong to two scientific fields that have been traditionally widely separated, and research at their interface bears a number of challenges to overcome, both experimentally and culturally [1]. Nevertheless, in recent years a number of studies have allowed great progress in this research area, pushing the limits of what was considered possible, and demonstrating great innovation potential for a wide number of applications. Enzymes can be covalently conjugated onto nanocarbons, adsorbed on their surface, or even encapsulated in those with a hollow structure, as reviewed elsewhere [2,3,4,5]. The interactions between proteins and nanocarbons can play an important role in determining their fate in vivo, including their biodegradation [6,7,8], formation of a biocorona [9,10,11], and consequent modulation of the immune response [12].

Besides the exciting area of biointegration of nanocarbons, for instance for the regeneration of conductive tissues [13], and (flexible) bioelectronics’ development [14], the fields of sensing and diagnostics [15] certainly benefit from the inclusion of both nanocarbons and enzymes. Finally, considering the current emergency we are facing in terms of environmental impact of human activities and climate change that requires urgent action, the use of enzymes is very attractive for the production, modification, and degradation of nanocarbons to render the relevant processes more sustainable.

To provide a general overview of the diverse members of each family, this minireview will briefly introduce the classification of enzymes and nanocarbons, followed by an overview of the literature describing their combination. We will then discuss the recent progress in a variety of applications, focusing on the last five years, and directing the reader to reviews of each topic where older research can be found. Finally, we will conclude with a perspective towards the future of this exciting research area.

### 1.1. Enzyme Classification

**Figure 1:**
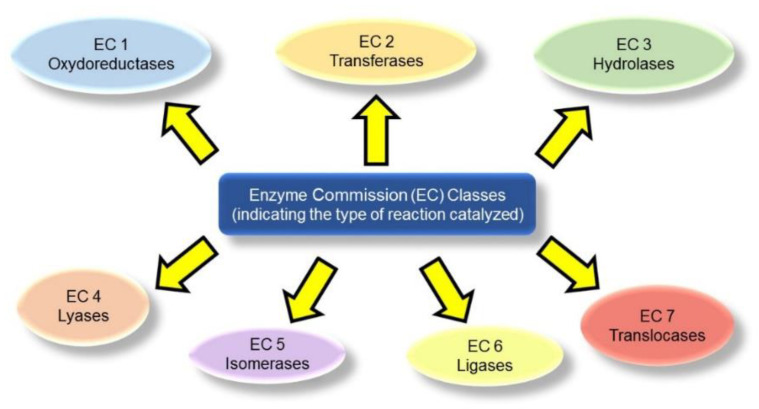
Seven enzyme classes constituting the first component of the Enzyme Commission (EC) number.

Enzymes have attracted scientists’ attention for a long time. It is imperative to adopt an unambiguous nomenclature and classification for this enormous and diverse group of biological catalysts to permit their rigorous study and accelerate scientific progress in this area. The International Union of Biochemistry and Molecular Biology (IUBMB) has introduced the IUBMB ExplorEnz website [16], where it is possible to navigate the Enzyme Commission (EC) system, which uses four-component numbers to identify each enzyme (i.e., EC X.X.X.X):

1. The first component refers to the general type of reaction being catalyzed. For instance, EC 1 indicates oxidoreductases that catalyze redox reactions, and EC 3 identifies hydrolases that catalyze hydrolytic reactions (Figure 1).
2. The second number indicates the subclass based on the type of compound or functional group involved in the reaction. For example, EC 1.13 refers to oxygenases that insert oxygen on the substrate, and EC 2.3 indicates acyl-transferases that transfer acyl groups, etc.).
3. The third component denotes the sub-subclass, by further specifying the reaction being catalyzed, for instance in terms of acceptors, or specific groups being transferred. As an example, EC 2.1.1 indicates methyl transferases.
4. The fourth component is simply a serial number that refers to the specific enzyme.

However, the system presents room for improvement, because it is not always unambiguous. In fact, new classes are constantly being added to the EC system, and it should also be noted that non-physiologically occurring enzymes are not included [17]. For these reasons, many other databases can be useful to complement the ExplorEnz information. For instance, helpful information can be found in the relevant metabolic pathways described on KEGG [18], kinetic data on BRENDA [19], thermodynamic data on NIST [20], and human gene names curated by HUGO [21] and NCBI [22]. New databases are continuously being created, including some that use different classification criteria, such as MEROPS for peptidases [23], and others that more generally collect various types of data pertaining to proteins, such as Expasy [24].

### 1.2. Carbon Nanomaterials (CNMs)

**Figure 2:**
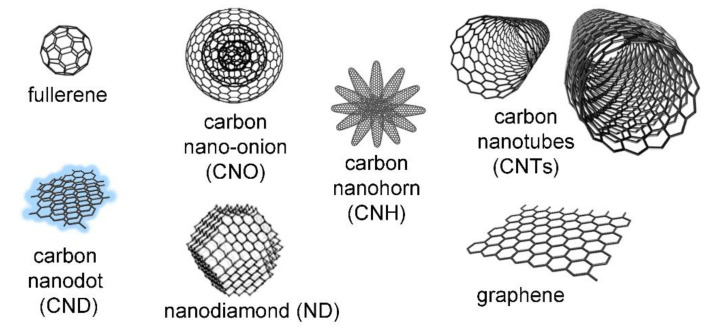
Main types of carbon nanostructures (not to scale). Reproduced from [26] under a Creative Commons license. The CNO schematic structure is adapted with permission from [27], copyright ©1996, Elsevier.

The family of CNMs is very diverse, with new members continuously being discovered. In this minireview, we will examine the most popular types of CNMs (Figure 2), as well as their general properties. Those features are briefly discussed in the following sub-sections, where readers are referred to reviews that describe in more depth the various CNM types that are mainly composed of carbon atoms (Figure 2), and their general properties. We will briefly mention their main features in the following sub-sections, where readers are referred to recent reviews that describe more in depth the various CNM types [25].

Generally, sp^2^-hybridized carbon allotropes share common features, such as high mechanical strength and electronic conductivity, together with low density, and a high surface area; these can be functionalized to tailor their properties ad hoc for the intended application, as reviewed elsewhere [15,28]. In particular, the various modes of interaction and conjugation with proteins is a widely covered topic in the literature, and thus it will not be discussed here [1,2,29]. CNMs composed mainly of sp^3^-hybridized carbon atoms exist also, such as nanodiamonds (NDs) [30]; they find scope especially in sensing and catalysis [31], although they have not attracted scientists’ attention to a great extent, likely due to their more limited accessibility relative to other CNMs.

Each carbon nanostructure has a specific size, morphology, curvature, and reactivity. However, despite the many research efforts on their modification for tailored applications, it is not always straightforward to predict a priori which one will give the best performance, with great variation across allotropes [32,33,34,35,36]. Nevertheless, their conductivity is particularly promising to enhance (bio)materials’ properties [37,38,39,40], for the regeneration of conductive tissues such as the challenging nerve [41,42,43,44,45] and cardiac tissues [46,47,48,49,50,51], in sensing [52] and various types of catalyses [53,54,55], and in the field of energy sustainable production [56], conversion [57], and storage [58]. Furthermore, the performance in the latter fields can be enhanced by the generation of highly porous materials. Indeed, carbon foams [59] and porous carbon [60] are highly advantageous since they can be produced at low cost and from a variety of precursors [61], such as polymers [62] and biomass [63], bearing high potential especially in gas [64,65,66] and energy storage [67,68,69,70,71], and in catalysis [72,73,74]. However, in this review we will focus on the traditional CNM types, as described in the following sub-sections.

#### 1.2.1. Fullerenes

Buckminsterfullerene, a discrete molecule composed of 60 carbon atoms (C_60_), was the first nano-allotrope to be discovered, in 1985 [75]. Fullerenes can also be composed of more carbon atoms, such as C_76_, C_84_, and C_90_ [76]. After C_60_, the rugby-ball shaped C_70_ is the second most known, and it is easier to isolate in sufficient amounts for research than higher fullerenes [77]. The advent of 100-milligram scale preparation of C_60_ [78] opened the way to decades of research that have provided several routes for its chemical functionalization, as recently reviewed [79]. Further tailoring of C_60_ properties can be attained through doping with heteroatoms [80]. Molecular confinement also offers the possibility to encase other elements in their interior to provide endohedral fullerenes [81] with interesting properties [82].

Fullerenes are fascinating molecules that have attracted researchers’ interest as they can be formed in space [83]. On Earth, they have been proposed for various applications, including the targeted delivery of therapeutics [84] and innovative antiviral therapies [85]. Despite the many potential uses in medicine [86], especially in photodynamic cancer therapy thanks to their ability to generate and modulate reactive oxygen species (ROS) levels [87], their electronic properties thus far found translation mainly in the field of photovoltaics, thanks to their electron-acceptor nature [88] and electron-transport ability [89].

#### 1.2.2. Carbon Nano-Onions (CNOs)

Multiple fullerenes can be organized one inside another in carbon nano-onions (CNOs) [27]. These concentric, multi-layered fullerenes can have a size ranging from 2 to 100 nm, depending on the method of synthesis [90]. Generally, multi-fullerenes display a decreasing reactivity with increasing size, corresponding to decreasing curvature, and therefore, the associated strain on the CNO surface. Consequently, small CNOs present good reactivity, although it should be noted that also other factors, such as the production method and the consequent amount of defect sites, affect their reactivity [91].

In the last decade, their biological [92,93] and electrochemical [94,95] applications have significantly expanded due to the favorable properties of the nanomaterial, including their small size, large accessible surface area, and high biocompatibility [96,97,98], especially once they are rendered soluble through covalent and non-covalent functionalization [99,100,101,102,103]. It is worth noting that recent findings described white-light luminescence arising when CNOs were produced through pyrolysis and underwent oxidative treatments [104].

#### 1.2.3. Carbon Nanohorns (CNHs)

Carbon nanocones [105] can form clusters termed carbon nanohorns (CNHs) [106]. Like many other CNMs, pristine nanocones unfortunately tend to aggregate in many solvents, and they do so in various morphologies termed dahlia-, bud-, or seed-like CNHs [107]. Despite this limitation, they can find promising applications in biosensing [108], medicine [109], and electrocatalysis [110], sometimes even outperforming other CNMs [111], and with relevance to clean energy [112] and carbon dioxide fixation [113]. There are not many studies on CNHs relative to the other CNMs, possibly also due to a more limited number of commercial sources. As a result, this type of nanomaterial remains an underexplored opportunity for innovation in numerous fields.

#### 1.2.4. Carbon Nanodots (CNDs)

In the last few years, luminescent carbon dots (CNDs) have emerged for their innovation potential, especially in sensing and biomedicine [114,115], thanks to their low-toxicity, chemical inertness, ease of preparation, environmental friendliness, and interesting physico-chemical properties [116,117]. Another key advantage relative to many other CNMs, is the excellent solubility of CNDs in a large variety of solvents, both organic and aqueous, depending on their chemical nature [118].

These nanomaterials have attracted great attention for their cost-efficient and sustainable production [119], using, for instance, natural products [120,121] or biomass waste as carbon source [122]. However, the fine tailoring of their desired optical properties has been challenging [123]. Indeed, the exact chemical structure of CNDs still poses many unanswered questions [124,125], and the thorough use of spectroscopic methods is key to providing an accurate characterization of the emittive species [126]. As we advance our understanding of CNDs’ nature, further prospects open up [127], expanding their applications to organocatalysis [128], sensitizers for photocatalysis [129] and pollutant degradation [130], and to energy conversion and storage [131], as capacitor electrodes, for instance [132].

#### 1.2.5. Nanodiamonds (NDs)

NDs are characterized by sp^3^-hybridization of their carbon core. NDs come in different sizes, morphologies, and surface types, depending on the method used for their preparation and functionalization [133]. They display attractive physico-chemical properties such as hardness, biocompatibility, and chemical inertness, leading to research on a variety of potential biological uses, especially delivery of therapeutics and imaging [134,135], but also sensing [136], tissue regeneration [137], skin products’ formulations [138], and in polished or active coatings with antimicrobial, antifriction, and mechanical reinforcing properties [139].

Recently, NDs have been considered also for their use in theranostics as applied to neurodegenerative diseases, thanks to their additional benefit of crossing the blood-brain-barrier [140]. Other emerging areas of application include primarily biological use in cells [141,142] and in vivo [143], but also catalysis [144].

#### 1.2.6. Carbon Nanotubes (CNTs)

Carbon nanotubes can be composed of one graphitic layer rolled up in single-walled CNTs (SWCNTs) [145], or multiple coaxial graphitic nanotubes called multi-walled CNTs (MWCNTs) [146], which can be grown with branches also [147]. CNTs can have very different properties; for instance, they can be semiconducting or metallic, depending on their type and chirality [148]. Over the years, several functionalization strategies have been developed [149]; oxidation is by far the most popular way to increase their polarity and dispersibility in various solvents, including water [150]. Therefore, fine control over their synthesis, purity and, thus, homogeneity is critical to enable their translation into large-scale use [151] and to fill the gap between their properties as individual CNTs and those of their bundled aggregates—often a practical limitation for industrial use [152].

CNTs have attracted great interest although there are still challenges to overcome to enable a wider commercial use of their unique properties [153]. Key areas of application include various types of high-performing composite materials, where demands of conductivity, robustness, flexibility, and mechanical resistance are high [154]. These include artificial neuromuscular prostheses [155,156], and more generally nano-bioelectronics [157] and wearable electronics [158], but also sensing [159] and imaging [160], orthopedic devices [161], tissue regeneration and biomedical use [162,163,164], electroactive materials for environmental and energy technology [165,166,167,168,169,170,171], electronics and computing [172,173], and various forms of catalysis [174,175,176,177,178,179].

#### 1.2.7. Graphene (G) and Graphene-Based Materials

In the last decade, the most popular carbon allotrope has been graphene (G), which can be considered as a 2D layer of sp^2^-hybridized carbon atoms arranged in a honeycomb lattice. It is worth noting that G can come in many forms, in terms of size, layers, level of oxidation, etc. which will all affect its physico-chemical properties [180]. In particular, graphene oxide (GO) [181,182] or its reduced form (rGO) [183,184] are often used for their improved dispersibility, relative to pristine G. G properties have also been tailored through topology [185], such as twists and nanoribbons [186]. Given the large heterogeneity in size, number of layers and of defects of G flakes, the general term of graphene-based materials is preferred over just G, to refer to this sub-class of CNMs [187].

Applications are similar to CNTs, and they include composite reinforcement [188], wearable [189] and flexible electronics [190,191], including memory devices [192] and even stretchable batteries [193], energy storage [194] and conversion [195,196,197,198], environmental remediation [199,200], varying types of catalysis [201,202,203,204], and innovative uses in the healthcare sector [205], such as regenerative medicine [206] and sensing [207,208]. In this case, large-scale, cost-effective production of high-quality G [209,210] and standardization are key for the translation of G properties into commodity products at a global level [211].

### 1.3. Bibliometric Analysis of CNMs and Enzymes

**Figure 3:**
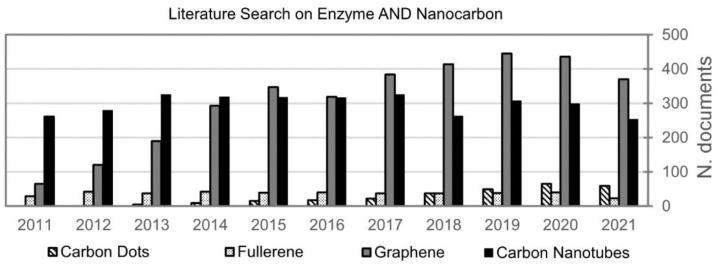
A literature search on carbon nanomaterials and enzymes focused on the last decade (Source: Scopus 14 November 2021).

A literature search for the term “enzyme” in conjunction with each one of the most popular CNMs shown in Figure 2, in the title, abstract, or keywords, has revealed that the vast majority of scientific articles pertain to CNTs (4.3 × 10^3^ documents), followed by G (3.4 × 10^3^ documents). However, in the last decade (Figure 3), scientific works on either one averaged about 300 per year, with a slight decrease for CNTs after 2017, opposed to a continuous increase for G up to 2019, surpassing CNTs in 2015. This trend could be related to concerns over CNTs’ toxicity, as suggested by the fact that scientific documents on this topic peaked at nearly 300 in 2015, and since then held steady. However, given their high innovation potential in medicine [212,213], and the high number of variables that affect their biocompatibility [214,215], alarming generalizations to ban their use are best avoided [216].

In comparison to CNTs and G, all the other CNMs lagged behind, each one reaching far less than 100 in total—with two exceptions—and representing today a missed opportunity for research. The first exception was fullerenes, possibly since they were the first to be discovered and thus have had more years of related research, reaching just over 600 records. The second exception was CNDs, which are among the most recent CNMs to be discovered. Scientific papers on CNDs have been increasing steadily year after year, since the first ones appeared in 2004. Therefore, it is foreseeable that CNDs will keep rising in popularity in the immediate future, although there is still a long way ahead to approach the numbers seen for CNTs and G.

In terms of applications, most studies pertain biosensing and biofuel cells (Table 1), although biocatalysis and biomedical applications other than sensing have been pursued with these systems. Interestingly, CNMs have also been envisaged as enzyme mimics, inhibitors, or detectors. We will concisely cover the progress made over the last five years on all these topics in the following sections.

## 2. CNMs for Enzyme Mimicry, Inhibition, or Monitoring

### 2.1. CNMs for Enzyme Mimicry

**Figure 4:**
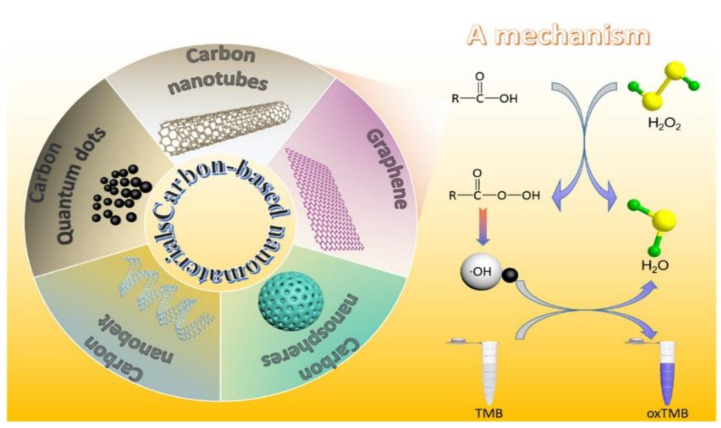
CNMs typically used for peroxidase mimicry (left) and a possible reaction mechanism that ultimately generates hydroxyl radicals for the oxidation of colorless 3,3′,5,5′-tetramethylbenzidine (TMB) to a colored product (oxTMB). Reprinted with permission from [300], Copyright © 2022, American Chemical Society.

A plethora of works describe the use of CNMs as nanozymes, meaning nanostructures that mimic enzymes as they display catalytic activity [289]. Research in this area is intended to overcome some of the common limitations of enzymes, particularly, the limited physico-chemical resistance against solvents and changes in temperature, pH or other experimental conditions [290]. Potential applications range from various biomedical applications [291], including innovative therapy [292] biosensing [293,294], and disinfection [295], to environmental monitoring and remediation [296]. In particular, peroxidase mimicry by CNMs (Figure 4) has been widely studied [297], especially for the development of glucose biosensors [298], although hydrolase mimicry is also attracting increasing interest [299].

In particular, CNDs have been functionalized with Fe (III) to mimic peroxidases and exert antimicrobial activity through generation of hydroxyl radicals [301]. In contrast, no hydroxyl radicals were generated when they were derivatized with glucose or cyclodextrin to mimic peroxidases, indicating a different mechanism [302]. The peroxidase mimicry activity can be correlated to the phosphorescence quantum yield and can inhibit bacterial growth under light irradiation, an activity that was envisaged for photodynamic antimicrobial chemotherapy applications [303]. Alternatively, the use of light could trigger radical oxygen species generation by the CND nanozymes, in an effort to mimic nuclease activity and cleave DNA [304].

Chemical functionalization has been successfully employed to attain CNDs with switchable fluorescence too. In this case, the fluorescence of amino-derivatized CNDs can be quenched by chelation with Fe(II) ions as nanozymes, and restored upon treatment with hydrogen peroxidase with a concomitant shift from yellow to green [305]. Furthermore, addition of other divalent metal cations can lead to additional advantages. For instance, Mn(II) extends the peroxidase mimicry by CNDs to neutral pH values, which is otherwise rather uncommon [306]. Despite the fact that mimicking enzymes’ enantioselectivity is a grand challenge, recent reports are demonstrating it is possible, in topoisomerase mimicry, for example [307]. Heteroatom-doped CNDs have been developed for theranostics as well, thanks to nanozyme activity [308]. Many other examples of nanozymes based on CNDs have been reported, through addition of other components, such as hemin [309], metal nanoparticles (NPs) [310,311], co-doping with various elements [312,313,314], MOFs [315], carbon nitride [316], and metal oxides [317].

Both graphene oxide (GO) and its reduced derivative (rGO) have also demonstrated peroxidase mimicking ability, which has been ascribed to the presence of carbonyl groups on the surface of the nanomaterial that get activated by hydrogen peroxide as a key step in the catalytic cycle [318]. Interestingly, rGO co-doping with N and B allowed development of nanozymes for the selective mimicry of peroxidases (but not oxidases) with enhanced catalytic performance for the development of biosensors [319].

**Figure 5:**
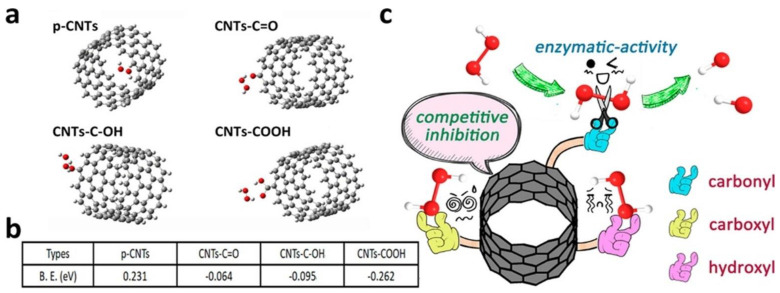
(**a**) Optimized binding modes between hydrogen peroxide and CNTs either in their pristine form (p-CNTs) or with different oxygen-bearing functional groups. (**b**) Binding energies between hydrogen peroxide and the various CNT types shown in (**a**). (**c**) Schematic illustration of peroxidase mimicry by oxidized CNTs. Reprinted with permission from [320], Copyright © 2022 American Chemical Society.

Analogously, oxidized CNTs demonstrated peroxidase-like activity, which was envisaged for the treatment of bacterial infections [320]. Different oxygen-bearing functional groups exert competing interactions with hydrogen peroxide (Figure 5), and thus control over oxidation is important [320]. Combination of CNTs with other chemical components is a popular strategy to tailor nanozyme activity to the intended application. In a recent example, SWCNTs have been functionalized with a nickel complex for the biomimicry of oxidase for H_2_ oxidation, and subsequent integration in fuel cells [267]. Alternatively, MWCNTs were coated with polypyrrole to introduce N-based ligands for Fe to be used in single-atom catalysis as peroxidase mimics [321]. CNTs were combined with hemin for peroxidase mimicry also [322,323]. They have been derivatized with polyoxometallate-based metal-organic frameworks (MOFs) for the selective sensing of cysteine [324], or with copper complexes [325], MOFs [326], and NPs [327] to develop nanozymes. For this type of application, many types of metal NPs have been used [328,329,330], as well as polymers to mimic phosphodiesterases [331].

CNTs can be further assembled into macroscopic materials, such as carbon nanofibers (CNFs), which find many uses, especially in high-performance composites and energy devices [332,333,334,335,336]. In this case, they have been decorated with Fe(III) complexes to mimic oxidases [337], peroxidases, and catalases for sensing and environmental technology [338]. Analogously to CNTs, CNFs can be oxidized, although gas-phase methods are preferable to the liquid-phase methods typically used for CNTs, to preserve the CNF macroscopic morphology, with the additional advantage of being virtually waste-free [339].

Peroxidase mimicry can be exerted by other CNMs as well, and for various uses. CNHs have been used as peroxidase mimics for the detection of drug traces as environmental pollutants [340]. They have been combined with nanosized ceria to detect hydrogen peroxide in commodity products, such as washing liquids and milk [341]. In the case of CNOs, nitrogen doping has been successfully applied to improve their catalytic performance in the electrochemical generation of molecular oxygen from hydrogen peroxide [342]. Boron and nitrogen co-doped CNOs showed great performance as electrocatalysts for the oxygen reduction reaction [95]. Interestingly, in the case of NDs, they were envisaged for redox-enzyme mimicry, with an activity that could be selectively tailored depending on the pH. At acidic pH, NDs catalyzed the reduction of molecular oxygen and hydrogen peroxide. At alkaline pH, they catalyzed the dismutation decomposition of hydrogen peroxide to produce molecular oxygen. It was proposed that the molecular mechanism of their peroxidase-like activity is electron-transfer acceleration, the source of which is likely derived from oxygen-containing functional groups on their surface [343].

Finally, besides peroxidases, and, generally, redox-active enzymes, which represent the vast majority of nanozyme mimicry studies on CNMs, hydrolases have started to attract scientists’ attention. In a recent report, fullerene derivatives were applied to this end through the presentation of multiple functional groups inspired from the natural enzymes’ catalytic sites [344]. Analogously to the other CNMs, fullerenes could also act as peroxidase mimics at acidic pH, and were thus envisaged for the eradication of *Helycobacter pylori* in vivo [345].

### 2.2. CNMs as Enzyme Inhibitors

**Figure 6:**
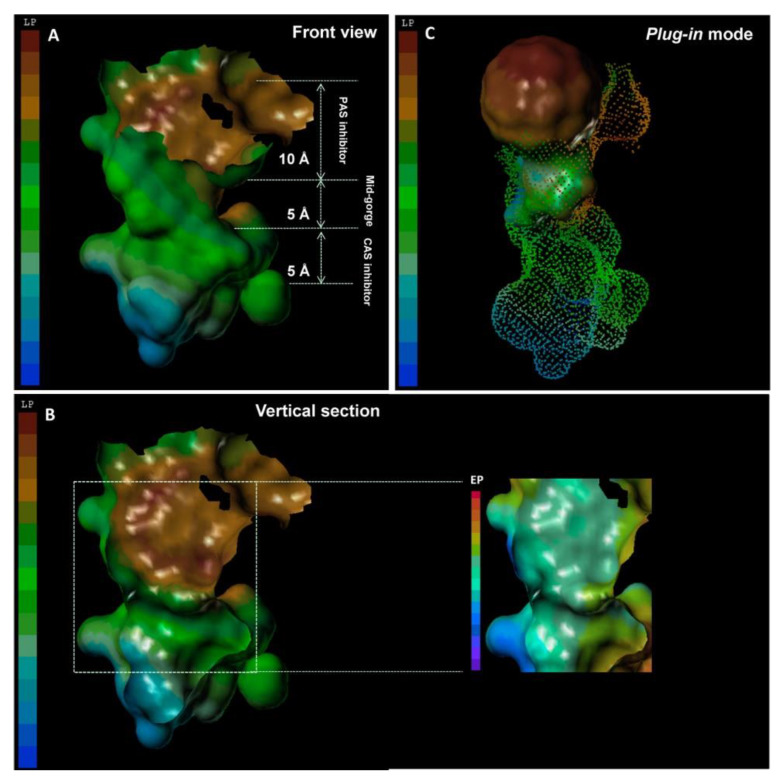
Front-view (**A**) and vertical section (**B**) of the surface of the active pocket of acetylcholinesterase, with the peripheral anionic site (PAS) giving access, through a narrow gorge, to the catalytic active site (CAS). (**C**) Fullerene (brown sphere) can interact with the PAS through hydrophobic interactions with the enzyme surface, whose lipophilic potential (LP) is color-coded from brown (highest hydrophobicity) to blue (highest hydrophilicity). Reproduced with permission from [349], Copyright © 2022, American Chemical Society.

Fullerene derivatives have been envisaged for the inhibition of a variety of enzymes, including recent examples of HIV-1 protease [346], ribonuclease A [347], glycosidases [347], ubiquitin-activating enzyme 1 [348], and acetylcholinesterase [349]. Their size, hydrophobic nature, and spherical morphology appear very suitable for hydrophobic interactions with lipophilic sites on the target enzymes (Figure 6), whilst C_60_ functionalization can add hydrophilic appendages for more specific interactions.

CNDs have been used to inhibit tyrosinase for cosmetic and food applications, thanks to hydrophobic interactions between the CNDs and the enzymatic surface, as well as chelation by the CND COOH groups of the enzyme copper ions. Tyrosinase is involved in the browning process of fruits and vegetables, and its overexpression has been linked to skin pigmentation disorders and tumorigenesis. Therefore, its inhibition could find several useful applications [350]. Furthermore, CNDs were found to tune glucose oxidase activity, depending on their functionalization type [224], and inhibit maltase, an effect that was envisaged as an innovative means to control physiological glucose levels [230].

Enzyme inhibition has been studied for CNTs also. SWCNTs demonstrated the ability to act as competitive inhibitors for proteases, such as chymotrypsin, thanks to hydrophobic interactions between the curved CNT surface and a morphologically complementary crevice on the enzyme surface, without alteration of the enzyme secondary structure or active site [351].

### 2.3. CNMs for Enzyme Monitoring

CNMs can be engineered to monitor enzymatic activity. Several examples have been reported especially using CNDs, whose fluorescence is initially quenched through interaction with a second component, and then restored upon a chemical transformation triggered by the activity of the target enzyme [352,353,354]. To this end, graphene CNDs have been functionalized with a cobalt derivative to allow for redox-dependent fluorescence that can be used to detect alkaline phosphatase activity, in serum, through the dephosphorylation of a substrate on the CNDs that releases ascorbic acid, which restores fluorescence [352]. Through a similar principle, silver NPs have been applied to quench the fluorescence of CNDs, so that, in the presence of enzymatic activity that generates hydrogen peroxide as a byproduct (e.g., through an oxidase), the silver NP structure decomposes, and fluorescence is restored. Applications in the health sector were envisaged, in particular for the monitoring of relevant biomolecules, such as glucose or cholesterol that could act as substrates for the corresponding oxidase [353]. Alternatively, glycosidases could be monitored through a similar principle, by functionalizing the CNDs to favor interaction with *p*-nitrophenol, which is generated through enzymatic activity on a glycosylated derivative [354,355]. Another target enzyme was thioredoxin reductase, overexpressed in many cancer cells [356].

SWCNTs have been applied to enzyme biosensing. Recently, SWCNTs were coated with a peptide to develop a biosensor for trypsin detection in urine samples, exploiting variations in CNT near-infrared photoluminescence upon enzymatic degradation of the peptide coating [357]. In another example, CNTs were envisaged for applications in cancer diagnostics, through the detection of matrix metalloproteinase-7, which is overexpressed in cancer cells [358]. Finally, CNT-fibers have been used to develop highly sensitive (54 µA·cm^−2^·mM^−1^) photoelectrodes for the detection of NADH, which is a key cofactor in many biocatalytic processes; its quantification correlates to specific enzyme activity [359].

## 3. Applications of CNM-Enzyme Conjugates

### 3.1. Biosensing

Biosensors typically comprise three elements, which are: (1) an element for biological recognition, such as an enzyme; (2) a transducer, to convert energy from the biorecognition event into another form (electrical, thermal, optical, etc.); (3) a signal processing system for the response readout and/or recording [360]. Biosensors often rely on enzyme inhibition, thus being ideal to monitor inhibitors that are relevant to human health, such as drugs or pollutants [361]. Enzymes are ideal components for biosensing, thanks to high sensitivity, specificity, low cost, and accessibility [360]. Coupling a semiconductor to enzymes can be exploited in photobiocatalysis, which is inspired by natural photosynthesis, but does not necessarily involve light for activation [362]. In general, inclusion of nanomaterials allows for better performance in a variety of analytical parameters, such as sensitivity, detection limit, stability, and response rate [363].

In particular, CNMs are ideal supports especially for biosensors that require multiple layers of enzymes, but also for providing a good electronic contact through the layers and with the electrodes [364]. CNMs can be good active supports for oxidoreductases as they may facilitate electron transfer to enhance catalysis, whilst offering a high surface area for high-level loading of enzymes [365]. However, the occurrence of direct electron transfer (DET) is a matter of ongoing debate, depending on the type of enzymes under consideration, the accessibility of their redox-active site to the CNMs, and the type of direct or indirect contact between CNMs and enzymes [265]. The electronic properties of CNMs render them attractive building blocks for electrochemical biosensors, besides the more traditional optical alternatives [366,367].

**Figure 7:**
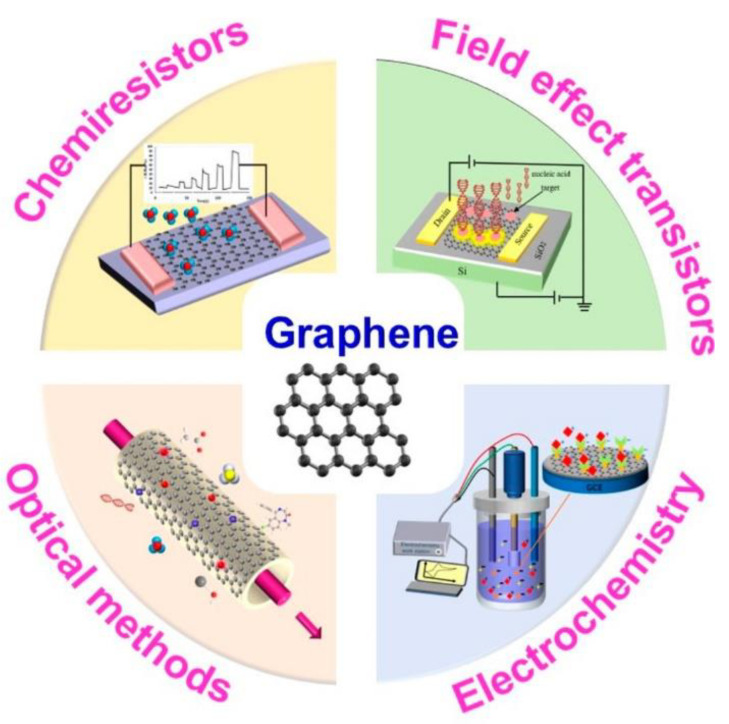
Graphene is one of the most popular CNMs, employed in a variety of biosensing devices thanks to its exceptional electronic and mechanical properties. Reproduced with permission from [368], Copyright © 2022 American Chemical Society.

Graphene is one of the most studied CNMs for a variety of biosensing devices (Figure 7) thanks to its exceptional electronic and mechanical properties, as recently reviewed in detail elsewhere [368]. Other less studied CNMs, such as CNOs, can also make attractive electrode components for the development of low-cost, simple to use, and highly sensitive sensors [237,369]. CNOs (mean size of 30 nm) were employed as electrochemical sensors by covalently immobilizing the glucose oxidase enzyme (GOx) on their surface via carbodiimide chemistry. GOx selectively catalyzed the oxidation of glucose, giving a sensor with high sensitivity and selectivity. However, the catalytic activity of GOx on the sensor electrode was highly sensitive to environmental conditions such as temperature, pH and humidity. Furthermore, the performance of the sensor was limited by enzymatic stability. Thus, an enzyme-free glucose sensor was designed, using Pt-decorated CNOs (Pt@CNOs) that outperformed many other CNMs previously studied for the same application [237].

**Figure 8:**
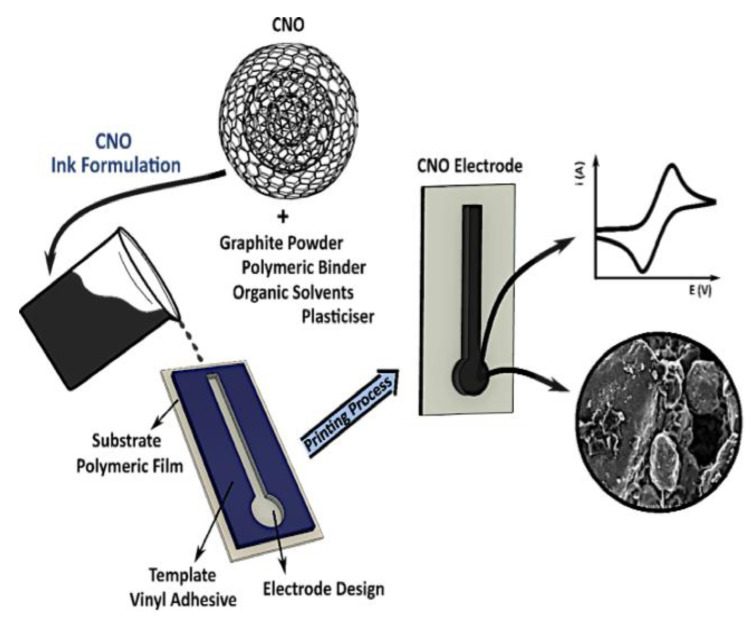
Screen-printed electrode preparation using a conductive ink based on graphite and CNOs. Reproduced from [369], under a Creative Commons license.

In 2020, Cumba et al. [369] described the preparation of the first ink that was based on CNOs to produce cheap and disposable electrodes, yielding sensors with elevated performance (Figure 8). Careful selection and optimization of all the components was a key step to attain a suitable formulation for the ink to be screen-printed. They included the conducting nanocarbons (i.e., graphite (GRT) and CNOs), the polymer binder, the plasticizer, and the organic solvent. The electrodes were screen-printed and they consisted of a conducting network of interconnected CNMs with a uniform distribution. The system displayed a heterogeneous electron transfer rate constant corresponding to 1.3 ± 0.7 × 10^−3^ cm·s^−1^ and also a current density that was higher than the ferrocene/ferrocenium coupled to a GRT screen-printed electrode that was commercially available. Furthermore, the CNO/GRT electrode allowed for the detection of dopamine in micromolar concentration (i.e., 10.0–99.9 µM), and with a 0.92 µM detection limit. The analytical sensitivity thus revealed a notable 4-fold increase relative to the commercial reference electrode based on GRT. Overall, this study opened the way to the use of CNO-based electrodes for high-performance sensing, electrocatalysis and battery research [369].

As can be seen from Table 1, the vast majority of CNM-enzyme conjugates have been studied for biosensing applications. The most popular target molecule is glucose for biometric health monitoring (Table 2) [224,227,228,237,249,269,271,273]. However, biosensors have been developed to detect many other bioactive compounds too, such as cholesterol [312,370,371] and triglycerides [282], lactose [220] and lactate [225,280], neurotransmitters [234,240,246,278,279,372] and hormones [239], various disease biomarkers [244,257,261], microRNAs [223], drugs [218,287], pathogens [219] and toxins [221], xanthine [262] and caffeic acid [373], *p*-coumaric acid [232], ferulic acid [233], trace metals [274], and oxygen [268].

### 3.2. Biofuel Cells

**Figure 9:**
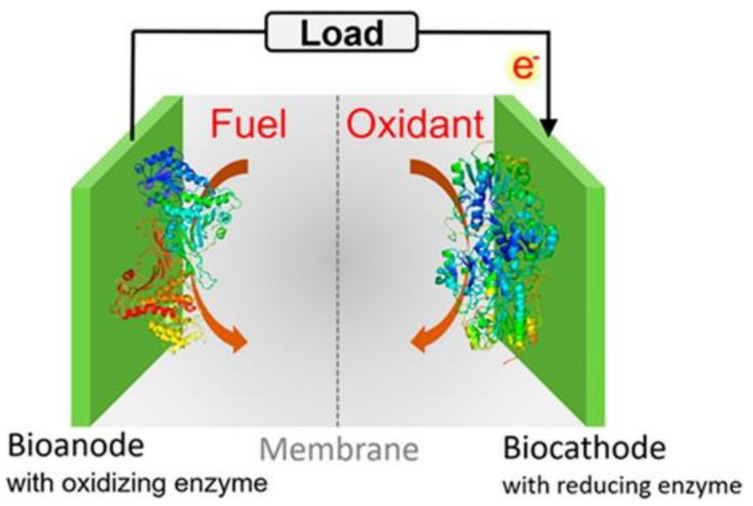
Schematic representation of a biofuel cells with enzymes at the bioanode, where the fuel is oxidized, and the biocathode, where oxidants are reduced. Reproduced with permission from [374], Copyright © 2022, American Chemical Society.

Biofuel cells are electrochemical devices that typically use redox enzymes as sustainable catalysts for the conversion of chemical energy into electrical energy (Figure 9); they consist of two-electrode cells that are separated by a proton-conducting medium. At the bioanode, fuels are oxidized, freeing electrons that flow to the biocathode through the external electrical circuit. At the biocathode, oxidants such as oxygen or peroxide are reduced to water [374]. Redox-active enzymes have attracted great interest for their use in the electrochemical production of fuels as sustainable alternatives in the field of clean energy, such as water splitting reactions [375].

Conjugation with CNMs allows for high-performance devices. They have been coupled to enzymes to serve as anodes [248,251,258,259,269,285], cathodes [253,267,276,277,286], or both [247,284].

An electrochemical reaction of particular interest is the molecular oxygen reduction (ORR) at the cathode. In this case, use of CNT-laccase as biocathode allowed reaching current densities >1.8 mA·cm^−2^, a direct electron transfer efficiency as high as 70–100%, and a turnover frequency of 5.0·10^3^ s^−1^ [253]. When bilirubin oxidase was used coupled to CNTs at the cathode, a maximum current density of 5.5 mA·cm^−2^ was found, and a power density of 1.85 mW·cm^−2^ at 0.6 V was attained, relative to 2.46 mW·cm^−2^ at 0.32 V with Pt/C as counter electrode [267]. Addition of catalase to a glucose oxidase (GOx)-CNT conjugate was thought to be another convenient strategy for ORR. In this case, GOx catalyzes the oxidation of glucose to gluconolactone with the concomitant consumption of molecular oxygen to produce hydrogen peroxide, which is then converted by the catalase into water and molecular oxygen that feeds back into the GOx reaction. As a result, this type of catalyst reached a maximum power density of 0.18 mW·cm^−2^ and a current density of 59 μA·cm^−2^ [276]. Another additive that can assist with catalytic performance in ORR is 2,2′-azino-di-(3-ethylbenzthiazoline sulfonic acid) or ABTS, which is a common substrate for hydrogen peroxidase and acts as an efficient electron transfer mediator between the enzyme and the electrode surface. With ABTS, a maximum power density of 1.12 mW·cm^−2^ at 0.45 V was obtained, which after two weeks had decreased just to 0.928 mW·cm^−2^, indicating good stability over time [277].

Wearable CNT-based biofuel cells were developed on a cotton textile that allowed illumination of an LED on the cloth [376]. Amongst the CNMs that have been used with enzymes in biofuel cells as summarized in Table 1, CNTs are certainly the mostly studied [247,248,251,253,258,259,267,269,276,277,284,285,286]. Recently, scientists are recognizing innovation opportunities also in other types of CNMs, such as CNDs [229], GO [242] or rGO [275], although reports in this direction are still very limited.

### 3.3. Biocatalysis

Thanks to great progress on biotechnology and protein engineering, biocatalysis has emerged as a green solution to increase the efficiency of industrial processes in a sustainable way [377]. Its importance and societal impact has been recognized through the Nobel Prize in chemistry in 2018 to Arnold, who pioneered the directed evolution that enabled development of resistant enzymes of industrial interest [378].

CNMs can be envisaged as active supports to immobilize enzymes and facilitate their recycling [379]. Besides, their electronic properties may favor the catalytic performance of redox-active enzymes. To this end, enzymes have been coupled to CNTs to enable asymmetric hydrogenation in flow [380]. Furthermore, bioelectrocatalysis involving direct electron transfer (DET) can benefit from the use of CNMs as active supports for redox enzymes, and the role played by their surface functionalization in the process has recently been reviewed [381].

### 3.4. Water Remediation and Environmental Monitoring

Enzymes supported on nanomaterials can be very convenient to detect pollutants for environmental monitoring through the development of sensitive sensors, but also for their removal from polluted waters [382]. For example, CNMs coupled to enzymes can be applied for the electrochemical monitoring of chromium [383]. CNDs’ fluorescence has also been envisaged for the optical detection of organic pesticides through coupling with an enzymatic reaction [384]. In addition, rGO has been envisaged for the detection of pesticides through the immobilization of an esterase on a biocomposite containing fibrin and thrombin, which was assembled taking inspiration form the blood coagulation process [245]. Finally, CNOs were coupled to a peroxidase in a cyclodextrin polymer matrix for the detection of herbicides, as tested in soil and river water samples [238].

### 3.5. Innovative Therapy and Theranostics

The rise of smart materials that can respond and adapt to stimuli and changes in the local microenvironment has opened new avenues that are enabling great progress especially in the biomedical field [385]. Enzymes can be used as convenient stimuli for the design of responsive materials [386], with great potential in the development of combined therapy and diagnosis, for instance through activation on a target pathological site characterized by the selective overexpression of certain enzymes [387]. The coupling of enzyme-responsive materials with nanostructures can be convenient to develop photodynamic therapies for cancer treatment [388].

Alternatively, enzymes can be supported onto CNMs for combined chemodynamic therapy (CDT). For example, MWCNTs were functionalized with Fe_3_O_4_ and glucose oxidase, so that the enzyme could convert glucose into gluconate and hydrogen peroxide. Conversion of the latter through the iron oxide-mediated Fenton reaction into hydroxyl radicals induces tumor cell death, and the reaction is favored by the lowered pH of the local microenvironment due to gluconate production. Finally, near-infrared (NIR) light irradiation can further boost the overall process at the target pathological site through generation of hyperthermia [252].

With the rise of biologics, enzymes have found applications also as therapeutic agents. As an example, laronidase can be used as replacement therapy for a type of mucopolysaccharidosis that is associated with deficiency of the natural enzyme, which hydrolyses glucosaminoglycans, causing their pathological accumulation in lysosomes. MWCNTs were thus envisaged as vectors for laronidase, which was covalently conjugated onto the CNMs [254]. There are clearly many unexplored opportunities in this research area that are worth future investigation.

## 4. Enzymatic Biodegradation of CNMs

**Figure 10:**
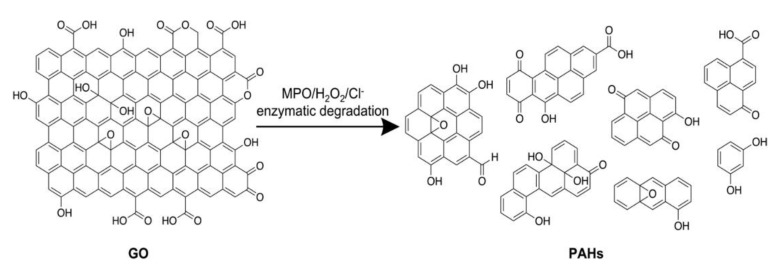
Enzymatic biodegradation of GO as a green production method of graphene quantum dots. Reproduced with permission from [389], Copyright © 2022, American Chemical Society.

The possibility of biodegrading CNMs through enzymatic activity is very appealing for various reasons, including lowering their persistence in the environment after use, but also avoiding or reducing their bioaccumulation in living organisms. Furthermore, the breaking down of larger CNMs, such as GO, into smaller components, can be envisaged as a green production method of graphene quantum dots (Figure 10) [389].

In general, the aromatic nature of CNTs renders them persistent in the environment, with little or no degradation by microorganisms [390], yet their oxidized forms appear to be biodegradable by microorganisms, whose enzymes are likely to use hydroxyl groups on the CNT surface as attackable sites that can be processed through enzymatic activity [391]. Various peroxidases have been found to be able to biodegrade CNTs and G derivatives, as recently reviewed [392]. They are mainly horseradish peroxidase (HRP), myeloperoxidase (MPO), manganese peroxidase (MnP) and lignine peroxidase (LiP). These four enzymes require hydrogen peroxide to participate in the degradation of CNMs. In the enzymatic degradation process of CNMs, molecular docking technology is used to predict possible binding sites, which helps to understand the degradation mechanism [393]. Recently, oxidases were reported to biodegrade MWCNTs [394], CNDs [8,395], and fullerenes [396]. It is not surprising to see that nanozymes are being developed for the same purpose, for instance as applied to the degradation of GO [397].

It is worth noting that besides the type of CNM, the level and type of functionalization is one of the factors playing a key role in determining the CNM biodegradation. Whilst it is accepted that oxidation generally favors biodegradation [150], other types of functionalization can have the opposite effect. In particular, chemical reduction of GO [398] and/or coating with bovine serum albumin or polyethylene glycol [399] rendered the CNM resistant to peroxidase-mediated biodegradation.

CNM biodegradation mediated by bacteria typically involves electron-transfer processes, which lead to the breaking of C–C covalent bonds. As a result, numerous pores arise on the surface of CNMs which lose structural integrity. Electrons can flow in either direction at the CNM-bacteria interface. In particular, cationic and anionic CNMs act as electron acceptors and donors, respectively [400]. Furthermore, oxygen interference can occur at the point of electron transfer between bacteria and CNMs [401]. In general, the functionalization of CNMs with anionic species on the surface of CNMs favors the electrostatic interaction with enzymes, which often display cationic amino acids on their surface, but also the catalytically-active heme group in redox-active enzymes plays a role in the interaction with CNMs. Clearly, pristine CNMs may be more challenging to degrade, and defect sites offer typical locations for the beginning of their structural deterioration [402].

Currently, fullerene biodegradation is still a largely unexplored research topic. It is known that this nanocarbon is challenging to degrade when exposed to soil bacteria [403]. However, the situation is notably improved in the case of organics-rich clay, such that more than half of the fullerene present can be mineralized just over two months, and even more so in the case of functionalized fullerol. Its structural deterioration can be notably accelerated through the combination of biodegradation with photochemistry, which likely mediates the destruction of the stable aromatic core [404]. Likewise, C_60_ photodegradation using UV light was facilitated by hydroxylation [405]. In another study, fullerene aggregates decreased in volume upon exposure to bacteria, with occurrence of hydroxylation, although the structural deterioration of the nanocarbon was slow and no significant production of carbon dioxide from C_60_ was noted, using isotope labelling [406]. In general, the efficiency of photodegradation can be relatively high, but it should be noted that only UV light can degrade CNMs. In natural environments, CNMs will react with other substances too, and their degradation by UV light will be affected by all these factors. There is still a knowledge gap in the detailed understanding of biodegradation of several CNMs, especially in realistic experimental conditions pertaining to those found in the environment, including soil and water.

## 5. Conclusions and Future Perspectives

Combining CNMs and enzymes requires a diverse skill set that is rare to find and represents a multidisciplinary research area that bears many technical and scientific challenges. However, a growing number of scientists are trying to innovate in this exciting field. The focus of our review has been to provide a concise overview from which it is evident how most studies have been focused on CNTs and, more recently, on graphene-based materials and CNDs, for applications in biosensing and biofuel cells. Nonetheless, CNMs offer far more benefits, and the multivarious members of the nanocarbon family still present today a valuable innovation opportunity that is worth exploring. Among other aspects that deserve further examination is their environmental fate, especially how biodegradation and photodegradation processes can improve the efficiency of CNM degradation.

Further research potential can be found in the development of computational methods to enhance enzymatic performance and robustness [407], including machine learning for enzyme engineering [408], potentially coupled to directed evolution approaches [409]. The range of enzymatic activity can be further expanded through the incorporation of unnatural amino acids [410], thanks to the emergence of robust methods for their genetic encoding [411]. Higher levels of complexity for the development of the next-generation devices can be attained with the incorporation of multienzymatic cascade reactions [412], also in confined environments [413], in an attempt to mimic, or go even beyond, the mesmerizing performance of biochemical cascades in living organisms. To this end, advancing electrochemical techniques for the characterization of enzymes at the electrode interface will be key [414], especially to leverage the unique electronic properties of CNMs and their application to further enhancing enzymatic activity. In particular, an attractive area is the development of wearable, flexible bioelectronics for the harvesting of bioenergy and its use in self-powered biosensing for health monitoring [415].

## Figures and Tables

**Table 1:** Scientific literature since 2017 on carbon nanomaterials and enzymes for various applications. C_60_ = buckminsterfullerene. CNDs = carbon nanodots. CNFs = carbon nanofibers. CNHs = carbon nanohorns. CNOs = carbon nano-onions. DHase = dehydrogenase. GO = graphene oxide. rGO = reduced GO. MWCNTs = multi-walled CNTs. SWCNTs = single-walled CNTs.

CNM Type	Conjugation	Enzyme	EC Class	Application	Ref.
C_60_	Non-covalent	Laccase	1	Water purification	[217]
	Covalent	Laccase	1	Water purification	[217]
		Tyrosinase	1	Biosensing	[218]
C_60_, MWCNTs	Non-covalent	Endonuclease	3	Biosensing	[219]
CNDs	Covalent	Galactosidase	3	Biosensing	[220]
		Quinolinate phosphoribosyl transferase	2	Biosensing	[221]
	Non-covalent	Adenylate kinase	2	Biocatalysis	[222]
		Endonucleases	3	Biosensing	[223]
		Glucose oxidase	1	Biosensing/therapy	[224,225,226,227]
		Glucose oxidase, peroxidase	1	Biosensing	[228]
		Laccase, NAD^+^-dependent DHase, alcohol DHase, aldehyde DHase, formate DHase	1	Biofuel cell	[229]
		Lactate oxidase	1	Biosensing	[225]
		Maltase	3	Therapy	[230]
		Old yellow enzyme	1	Biocatalysis	[231]
		Uricase	1	Biosensing	[225]
CNFs	Covalent	Laccase	1	Biosensing	[232]
	Non-covalent	Tyrosinase	3	Biosensing	[233]
CNHs	Covalent	Glutamate oxidase	1	Biosensing	[234]
	Non-covalent	Peroxidase	1	Biosensing	[235]
CNOs	Covalent	Alkaline phosphatase	3	Biosensing	[236]
		Glucose oxidase	1	Biosensing	[236,237]
		Peroxidase	1	Biosensing	[236,238]
	Non-covalent	Adenylate kinase	2	Biocatalysis	[222]
GO	Covalent	Laccase	1	Biosensing	[239]
	Non-covalent	Choline oxidase/acetylcholine esterase	1/3	Biosensing	[240]
		Rhamnosidase	3	Biocatalysis	[241]
		Adenylate kinase	2	Biocatalysis	[222]
		Glucose Oxidase	1	Biosensing/fuel cells	[242]
		Lipase	3	Biocatalysis	[243]
GO, MWCNTs	Non-covalent	Lactate oxidase	1	Biosensing	[244]
rGO	Non-covalent	Acetylcholine esterase	3	Biosensing	[245]
		Thrombin	1	Biosensing	[245]
MWCNTs	Covalent	Choline Oxidase	1	Biosensing	[246]
		Glucose oxidase	1	Biosensing/fuel cells	[247,248,249,250,251]
		Glucose oxidase	1	Cancer therapy	[252]
		Laccase	1	Biofuel cells	[253]
		Laronidase	3	Therapy	[254]
		Lipase	3	Biofuel cells	[255]
		Peroxidase	1	Sensing/membranes	[256,257]
		Pyranose oxidase	1	Biosensing/fuel cells	[258,259]
		Tyrosinase	3	Biosensing	[218,260]
		Uricase	1	Biosensing	[261]
		Xanthine oxidase	1	Biosensing	[262]
	Non-covalent	Alcohol dehydrogenase	1	Biosensing	[263,264]
		Alcohol oxidase	1	Biosensing	[265]
		Amino acid oxidase	1	Biosensing	[265]
		Amylase, lysozyme	3	Biocatalysis	[266]
		Bilirubin oxidase	1	Biosensing/fuel cells	[267,268]
		Choline oxidase	1	Biosensing	[265]
		Glucose dehydrogenase	1	Biosensing/fuel cells	[269]
		Glucose oxidase	1	Biosensing/fuel cells	[247,270,271,272,273,274,275]
		Glucose oxidase/catalase	1	Biofuel cells	[276]
		Glucose oxidase, laccase	1	Biosensing/fuel cells	[277]
		Glutamate oxidase	1	Biosensing	[278]
		Laccase	1	Biosensing	[279]
		Lactate oxidase	1	Biosensing	[280]
		Lipase	3	Biosensing/biocatalysis	[263,264,281,282,283]
		[NiFeSe]-hydrogenase	1	Biofuel cells	[284]
		Oxalate decarboxylase	4	Biofuel cells	[285]
		Peroxidase	1	Biosensing/fuel cells	[286,287]
		Pyranose oxidase	1	Biosensing/fuel cells	[258,265]
SWCNTs	Covalent	Tyrosinase	1	Biosensing	[288]
	Non-covalent	Choline oxidase	1	Biosensing	[265]

**Table 2:** Comparison of recent reports (since 2017) that described CNMs-based sensors for glucose detection.

CNM Type	Conjugation	Linear Range	Detection Limit	Sensitivity	Ref.
CNDs	Non-covalent	250–3000 μM	n.a.	n.a.	[224]
		0.1–500 μM	65 µM	21.6 µA·mM^−1^·cm^−2^	[227]
	Covalent	0.1–500 μM	0.04 μM	n.a.	[228]
CNOs	Covalent	1000–10,000 μM	210 μM	26.5 µA·mM^−^^1^·cm^−2^	[237]
CNTs	Covalent	100–6000 μM	9.01 μM	n.a.	[249]
		n.a.	n.a.	0.27–3.7 µA·mM^−^^1^·cm^−^^2^	[258]
	Non-covalent	1000–20,000 μM	n.a.	0.198 µA·mM^−^^1^·cm^−^^2^	[269]
		1–5000 μM	0.36 μM	n.a.	[271]
		0–5000 μM	50 μM	289 μA·mM^−1^·cm^−2^	[273]

## Data Availability

Not applicable.
